# The Clinical Effectiveness of Preimplantation Genetic Diagnosis for Chromosomal Translocation Carriers: A Meta-analysis

**DOI:** 10.1055/s-0040-1712455

**Published:** 2020-07-08

**Authors:** Manijeh Mahdavi, Seyedeh M. Sharafi, Seyede S. Daniali, Roya Riahi, Majid Kheirollahi

**Affiliations:** 1 Pediatric Inherited Diseases Research Center, Research Institute for Primordial Prevention of Non-Communicable Disease, Isfahan University of Medical Sciences, Isfahan, Iran; 2 Environment Research Center, Research Institute for Primordial Prevention of Non-Communicable Disease, Isfahan University of Medical Sciences, Isfahan, Iran; 3 Child Growth and Development Research Center, Research Institute for Primordial Prevention of Non-Communicable Disease, Isfahan University of Medical Sciences, Isfahan, Iran; 4 Department of Genetics and Molecular Biology, School of Medicine, Pediatric Inherited Diseases Research Center, Research Institute for Primordial Prevention of Non-Communicable Disease, Isfahan University of Medical Sciences, Isfahan, Iran

**Keywords:** pregnancy outcome, preimplantation genetic diagnosis, meta-analysis, translocation, recurrent miscarriage

## Abstract

Published data on the relationship between pregnancy outcomes of preimplantation genetic diagnosis (PGD) in translocation carriers have implicated inconclusive results. To identify potentially eligible reports, an electronic search was conducted in several databases, including PubMed, Scopus, Web of Knowledge, and Cochrane. Pooled odd ratios (ORs) and 95% confidence intervals (Cis) were estimated based on a random-effect model to evaluate the strength of association between PGD and successful pregnancy outcome in translocation carriers. A total of six cohort studies were included in the current study. The meta-analysis of these studies revealed that the PGD method was associated with an increased successful pregnancy outcome of translocation carriers (OR = 8.58; 95%CI: 1.40–52.76). In subgroup analysis, there was no significant association according to the chromosomal translocation carrier origin and the type of translocated chromosomes, as well as country. In developed countries, the pregnancy outcome of PGD was significantly improved in translocation carriers (OR = 21.79; 95%CI: 1.93–245.52). The current meta-analysis demonstrated that the PGD method is associated with successful pregnancy outcome in both types of reciprocal and Robertsonian translocation carriers, especially in developed countries.

## Introduction

Chromosome structural rearrangement, including reciprocal and Robertsonian translocation, is the most common type of chromosome abnormality. 1 It is the leading cause of implantation failure, infertility, recurrent miscarriage, and congenital abnormalities caused by an unbalanced karyotype in humans. 2 In these carriers, the segmental affinities between the translocated and normal chromosomal regions produces unbalanced rearrangements at high frequency, due to quadrivalent pairing rather than bivalent at meiosis. 3 Reciprocal translocations—typically as an exchange of two terminal segments from different chromosomes—are found in approximately one in every 500 live births, whereas Robertsonian translocations, the centric fusion of two acrocentric chromosomes, has a less prevalence in the population about 1 in 1,000. 4

Although there is a high probability for a successfully natural outcome in many translocations, patients carrying translocations with a significant risk of viable abnormality are increasingly pursuing to improve their chances of a normal pregnancy with the help of preimplantation genetic diagnosis (PGD). 5 PGD can select balanced embryos and avoid the transfer of embryos with unbalanced chromosomal rearrangements and thus reducing the risk of recurrent miscarriages or the birth of a child with chromosomal abnormalities. 6 7 8 9 10 11

In recent years, various studies indicated that PGD may play pivotal roles in increasing successful pregnancy outcome in translocation carriers. 12 13 14 Several studies concluded that after PGD, the spontaneous abortion rate was significantly reduced in translocation carriers. 15 16 Some studies, reported after PGD, stated that the chance of live birth is low for translocation carriers and natural conception will be a better option. 12 17 18 However, the results from these studies are inconsistent.

The major reason for using PGD in translocation carriers is the reduction of miscarriages and more live births by eliminating the transfer of abnormal embryos. However, there is insufficient evidence regarding the pregnancy outcome for translocation carriers who underwent PGD and those of non-PGD patients. The statement that PDG increases successful pregnancy outcomes should be confirmed before the technique is applied for daily clinical practice. To improve informed decision making, we conducted a meta-analysis regarding clinical effectiveness and pregnancy outcomes after PGD, in couples carrying translocation chromosomal abnormality in comparison to none-PGD group. Until now, no meta-analysis has been performed to investigate this purpose. The aim of the present study was to assess the outcome of PGD in couples who at least one partner is a carrier of a reciprocal or Robertsonian translocations.

## Methods

### Search Strategy

The databases PubMed, Scopus, Web of Knowledge, and Cochrane were systematically searched for all available articles published till 2018, without considering limitation for any age range, time, or language. Publications with the following search words in the titles, abstract, or keywords of the original studies were included: “clinical effectiveness” OR “outcome” OR “pregnancy outcome” AND “preimplantation genetic diagnosis” OR “PGD” OR “PGP” OR “preimplantation genetic profiling” AND “translocation” OR “Chromosomal translocation.” We also improved this search by reviewing the reference lists of all of the retrieved publications and identifying supplementary relevant articles.

### Inclusion and Exclusion Criteria

For our meta-analyses, articles with the following criteria were included 1 : (1) any study published as an original study that focused on the pregnancy outcome of PGD in translocation carriers 2 ; (2) the numbers of case and control groups for each PGD and non-PGD group were reported or the relevant data were available; and (3) sufficient data were provided to estimate the odds ratio (OR) and 95% confidence interval (CI). In addition, we excluded reviews, editorials, comments, case reports, and overlapped articles or studies with overlapping data and inadequate information for pregnancy outcome of PGD.

### Data Extraction

The articles were selected and extracted of the original data by two of the authors (M.M. and S.S.D.) independently using a standardized and consistent method. The following information was collected from each study: first author, year of publication, ethnicity of the patients, numbers of cases and controls, PGD method and variables adjusted for in the analysis, as well as multivariate adjusted ORs and 95% CIs.

### Statistical Analysis

The pregnancy outcome of PGD in translocation carrier populations was estimated by calculating pooled ORs and associated 95% CI. The significant of the pooled OR was determined by *Z* -test. All statistical analyses were conducted using STATA software (version 12.0; Stata Corp LP, College Station, Texas, United States) and *p* < 0.05 was considered statistically significant. To detect heterogeneity among studies, the Chi-square test–based Q statistic was performed and was quantified using the *I* ^2^ statistic. 19 When heterogeneity was significant ( *p* < 0.05 and *I* ^2^ > 50%), random-effect model (the DerSimonian–Laird method) was employed. 20 We conducted a sensitivity analysis to explore heterogeneity when significant heterogeneity existed. Subgroup analysis was applied by country, female/male carrier, and the type of translocated chromosomes. Furthermore, both Begg's and Egger's tests were performed to evaluate publication bias, *p* < 0.05 for these tests indicate significant publication bias.

## Results

### Characteristics of the Included Studies

**Fig. 1:**
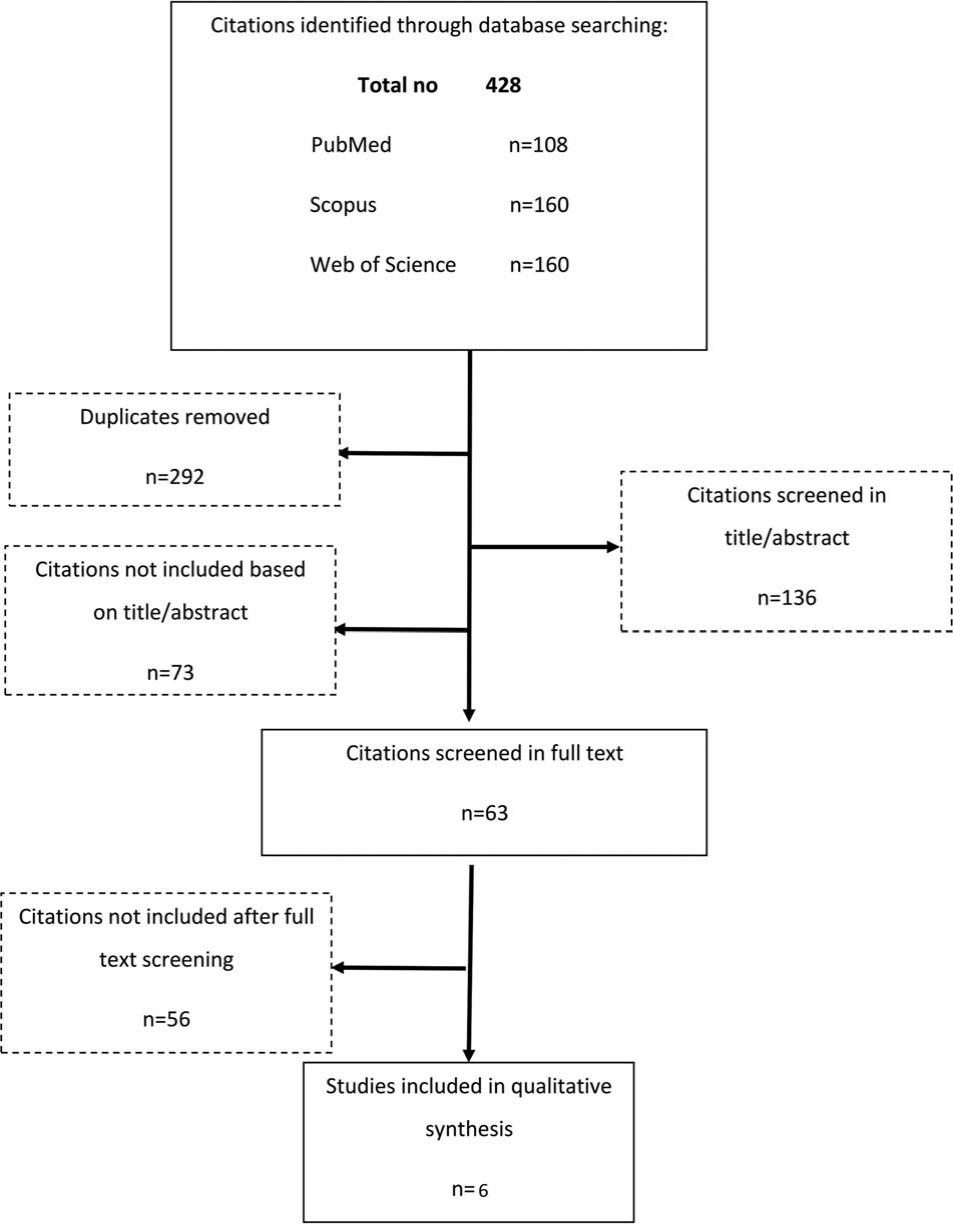
Flow chart of the study selection process.

A detailed ﬂow chart of the study selection process is shown in Fig. 1 . According to search, a total of 428 potentially relevant articles were identified. After removing duplicates, 292 publications were included for further evaluation. Among these articles, 63 articles were selected for reviewing the full text. Overall 55 publications were excluded mainly because of no relevance, animal not human experiments, reviews, or meeting abstract. One study was excluded for the reason that it was not possible to calculate OR. 21 At the final step, six full-text articles were included in the present meta-analysis. The main characteristics of included studies in the meta-analysis were summarized in Table 1 . As shown in the table, two studies involved Asian 15 17 and four involved developed countries. 16 18 22 23 Three studies focused on the female carriers of chromosomal translocations, 15 18 23 and the remaining three studies were associated with one of the partner male/female carrier. 16 17 22 All of the studies was evaluated the pregnancy outcome of PGD in both types of chromosomal translocations (Reciprocal and Robertsonian) except one study in reciprocal translocation carriers. 18 Translocated chromosomes 13 and 21 were analyzed by PGD in all of the studies. The other chromosomes, such as X and Y, were only studied in three papers. 18 22 23 The main PGD method was fluorescent in situ hybridization (FISH) which was used in all of the included studies.

**Table 1:** Characteristics of studies in the meta-analysis

ID	Study	Country	No. of cases	Year	Translocation type/chromosome	Translocation origin	Miscarriage rate	PGD method	Study design	Statistical analysis
1	Kyu Lim et al 15	South Korea	18	2004	Both Rcp and Rob/ Rcp (1–22,Y) Rob 13–15, 21	Female	3	FISH	Cohort	*p* = 0.02
2	Munné et al 16	USA	16	2000	Both Rcp and Rob/1–22	Male/female	2	FISH	Cohort	*p* = 0.001
3	Ikuma et al 17	Japan	37	2015	Both Rcp and Rob/1–22	Male/female	3.37 ± 1.26	FISH	Cohort	*p* = 0.101
4	Keymolen et al 18	Belgium	138	2012	Reciprocal translocation carriers (Rcp)/13, 18, 21, X and Y	Female	3–4	FISH	Cross-sectional	OR = 0.62 (CI: 0.16–2.31)
5	Verlinsky et al 23	USA	43	2005	Both Rcp and Rob/13, 15, 16, 17, 18, 21, 22, X, Y	Female	0–4 or more	FISH	Cohort	*p* = 0.001
6	Fischer et al 22	USA	69	2010	Both Rcp and Rob/X, Y, 8, 13–22	Male/female	3–7	FISH	Cohort	*p* = 0.0001

Abbreviations: CI, confidence interval; FISH, fluorescent in situ hybridization; OR, odds ratio; PGD, preimplantation genetic diagnosis; Rcp, reciprocal translocation; Rob, Robertsonian translocation.

### Meta-analysis Results

**Fig. 2:**
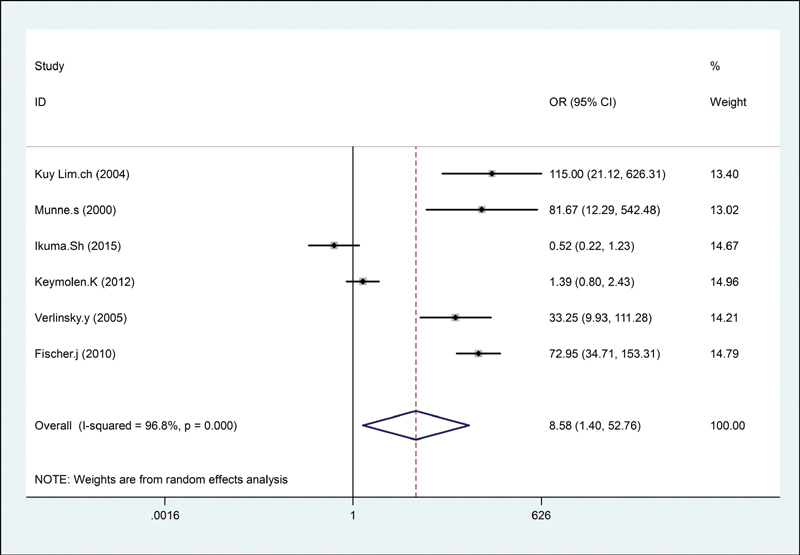
The forest plot of the meta-analysis for successful pregnancy outcome in translocation carriers. CI, confidence interval; OR, odds ratio.

**Fig. 3:**
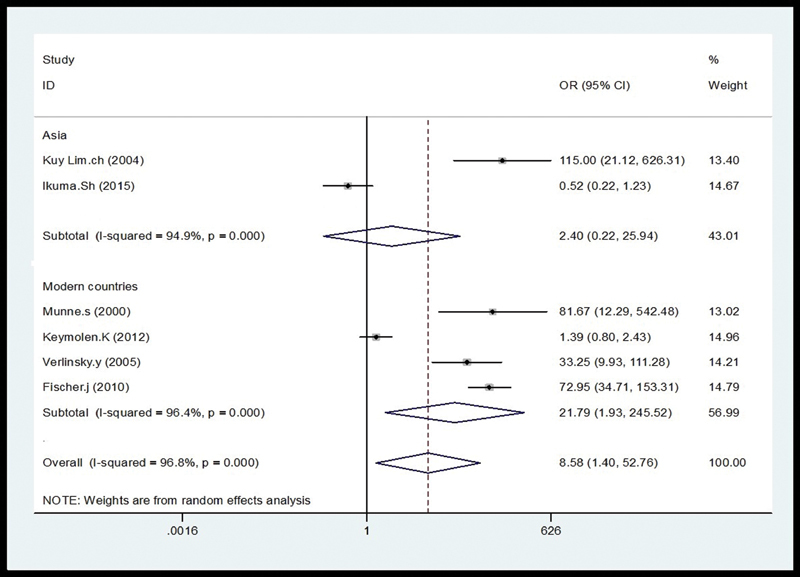
The subgroup analysis, according to country. CI, confidence interval; OR, odds ratio.

**Fig. 4:**
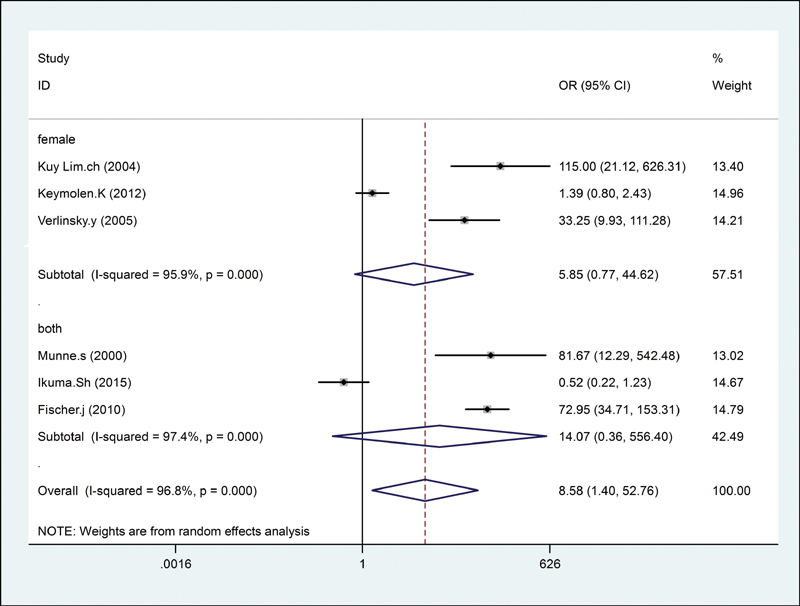
Origin of the translocation carrier. CI, confidence interval; OR, odds ratio.

**Fig. 5:**
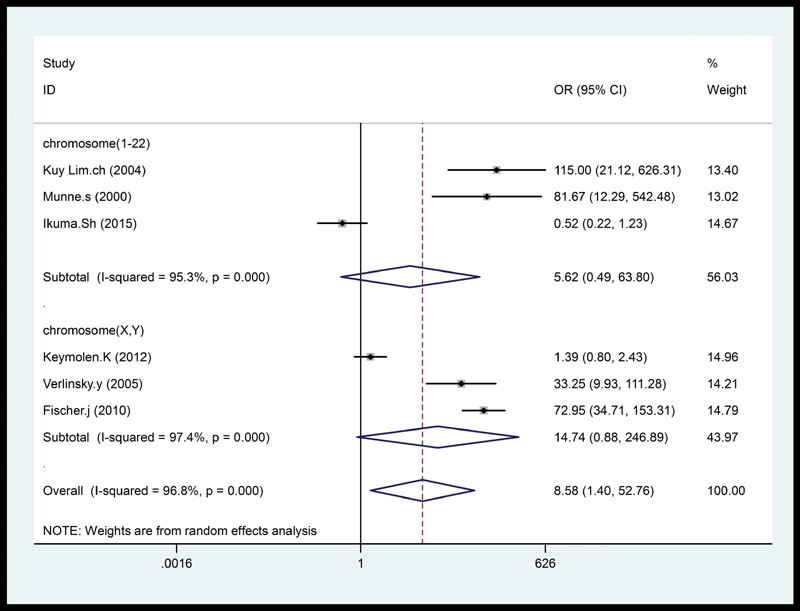
The translocated chromosome subgroup analysis. CI, confidence interval; OR, odds ratio.

The forest plot of the meta-analysis for successful pregnancy outcome in translocation carriers is shown in Fig. 2 . Overall, significant association was found between PGD and successful pregnancy outcome in the translocation carriers (OR = 8.58; 95% CI: 1.40–52.76; *I* ^2^ = 96.8%). For the subgroup analysis, according to country ( Fig. 3 ) PGD was consistently associated with increased successful pregnancy outcome in developed countries (OR = 21.79; 95% CI: 1.93–245.52; *I* ^2^ = 96.4%) but no significant association was found in Asian countries (OR = 2.40; 95% CI: 0.22–25.94; *I* ^2^ = 94.9%). Moreover, for the translocation carrier origin (male/female), significantly increased successful pregnancy outcome of PGD in translocation carriers was not found in female (OR = 5.85; 95% CI: 0.77–44.62; *I* ^2^ = 95.9%) and one of the partner carriers (OR = 14.07; 95% CI: 0.36–556.40; *I* ^2^ = 97.4%; Fig. 4 ). In the translocated chromosome subgroup analysis, there was no significant differences between PGD and pregnancy outcome in presence of translocation for sexual chromosomes X–Y group (OR = 5.62; 95% CI: 0.49–63.80; *I* ^2^ = 95.3%) and autosomal chromosomes 1 to 22 (OR = 14.47; 95% CI: 0.88–246.89; *I* ^2^ = 97.4%) group ( Fig. 5 ).

### Heterogeneity Test and Sensitivity Analysis

In the present meta-analysis, significant heterogeneity was observed. We next performed a sensitivity analysis by removing the individual studies sequentially to assess the effect of individual studies. The results showed that there was no different from the initial analysis (figure not shown), suggesting that the results of the meta-analysis were strong.

### Publication Bias Analysis

**Fig. 6:**
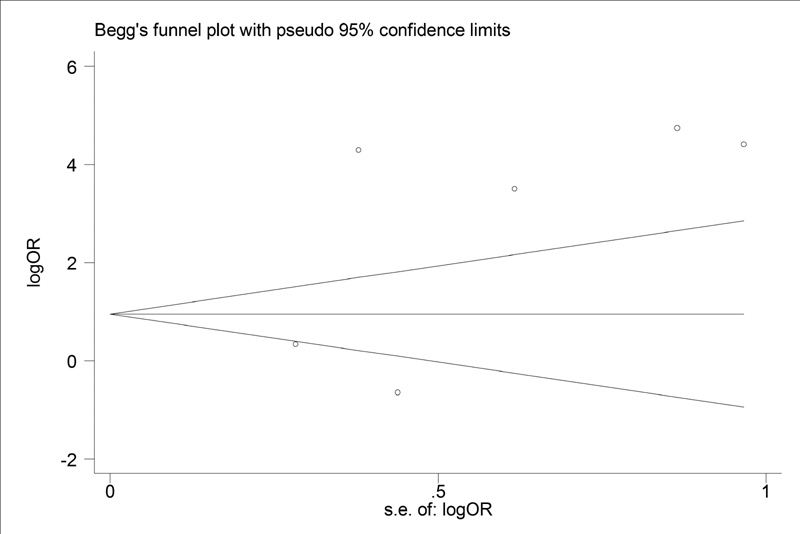
Funnel plot. OR, odds ratio.

Funnel plot and Egger's test were executed to access publication bias. Both funnel plots ( Fig. 6 ) and Egger's test ( *p* = 0.16) suggested no evidence of publication bias in the meta-analysis.

## Discussion

It is well known that chromosomal abnormalities, such as translocation, are of important reasons for recurrent miscarriage losses. PGD might seem an attractive alternative for couples carrying chromosomal translocation. 24 Although some studies pointed to the poor outcome, even after PGD, success rates of the pregnancies after proper therapeutic procedures in the couples with poor previous pregnancy results were confirmed by the majority of researchers. On the other hand, selection of cases to take PGD is the key of successful result, so that women with of advanced maternal age, infertility and having recurrent pregnancy loss (RPL) experience were recommended to successful result.

PGD analysis of translocations' embryos has been studied since the 1990s. 25 26 To our knowledge, this is the first comprehensive meta-analysis investigating the pregnancy outcome of PGD in translocation carriers. Recent systematic reviews of PGD for carriers of a structural chromosome with a history of recurrent miscarriage 14 and unexplained recurrent miscarriage 27 have not shown benefit with this strategy, compared with medical management. They concluded that there are insufficient data showing that PGD improves the live birth rate in couples with recurrent miscarriage carrying a structural chromosome abnormality.

Our meta-analysis illustrates strong evidence for significant association between the pregnancy outcome of PGD in reciprocal and Robertsonian translocation carriers. The findings from subgroup analysis indicated the significantly positive effect of PGD on pregnancy outcome in translocation carriers from developed countries not at Asian countries. However, Kyu Lim et al reported that spontaneous abortion rate could be significantly reduced by PGD in translocation carries from Korean population. 15 Furthermore, in the current study, the pregnancy outcome of PGD in translocation carriers was not depended on the carrier origin and the type of translocated chromosomes.

In Asian studies, all of the participants had only translocation with two or more consecutive clinical miscarriages, 16 21 while other studies in developed countries including all of infertility problems or RPL or still birth which may affect the overall outcome of their studies. Also the mean age of the patients who underwent PGD was significantly higher than control group in one of Asian studies, 16 while there wasn't significant difference between age of control and PGD group in other studies.

The clear evidence of heterogeneity in this meta-analysis should be discussed. Though a sensitivity analysis was performed, the origin of the heterogeneity among the studies was not found. The heterogeneity might have been due to other factors, such as diversity in the population characteristics (ethnicity, age, the type of translocation, etc.), PGD methods, and study design. Our meta-analysis was based on estimates without adjusting the data for these factors, which is the potential limitation of this study. Some another limitations of our meta-analysis was the insufficient number of studies, especially for subgroup analysis and languages of the publications.

## Conclusion

In conclusion, this meta-analysis provide reliable evidence that the PGD method is associated with the development of pregnancy outcome in translocation carriers, especially in developed countries. However, it is required to conduct further larger scale, multicenter, and high-quality studies in the future.
